# Incidence and impact of new-onset postoperative arrhythmia after surgery of the lower gastrointestinal tract

**DOI:** 10.1038/s41598-023-27508-4

**Published:** 2023-01-23

**Authors:** Felix Rühlmann, Mara Sophie Hedicke, Deborah Engelhardt, Alma Franziska Mackert, Tobias Tichelbäcker, Andreas Leha, Markus Bernhardt, Michael Ghadimi, Thorsten Perl, Azadeh Azizian, Jochen Gaedcke

**Affiliations:** 1 Department of General, Visceral, and Paediatric Surgery, University Medical Centre Göttingen, Robert-Koch-Str. 40, 37075 Göttingen, Germany; 2 Clinic III for Internal Medicine, Heart Centre of University Hospital of Cologne, Cologne, Germany; 3 Institute of Medical Statistics Göttingen, University Medical Centre Göttingen, Göttingen, Germany

**Keywords:** Cardiology, Medical research, Risk factors

## Abstract

Postoperative arrhythmias (PAs) are common events and have been widely investigated in cardiothoracic surgery. Within visceral surgery, a recent study revealed a significant occurrence of PA in esophageal resections. In contrast, PA in lower gastrointestinal surgery is rarely investigated and has been rudimentary described in the medical literature. The present work is a retrospective cohort study of 1171 patients who underwent surgery of lower gastrointestinal tract between 2012 and 2018. All included patients were treated and monitored in the intensive care unit (ICU) or intermediate care unit (IMC) after surgery. Follow-up, performed between January and May 2021, was obtained for the patients with PA investigating the possible persistence of PA and complications such as permanent arrhythmia or thromboembolic events after discharge. In total, n = 1171 patients (559 female, 612 male) without any history of prior arrhythmia were analyzed. Overall, PA occurred in n = 56 (4.8%) patients after surgery of the lower GI. The highest incidence of PA was seen in patients undergoing bowel surgery after mesenteric ischaemia (26.92%), followed by cytoreductive surgery (CRS) combined with hyperthermic intraperitoneal chemotherapy (HIPEC; 16.67%). PA was significantly associated with higher age (72 years (IQR 63–78 years) vs. 64 years (IQR 55–73.5 years), *p* < 0.001) and longer length of stay in the ICU (median 15 days (IQR 5–25 days) vs. median 2 days (IQR 1–5 days), *p* < 0.001). PA was independently associated with organ failure (OR = 4.62, 95% CI 2.11–10.11, *p* < 0.001) and higher in-house mortality (OR = 3.37, 95% CI 1.23–9.28, *p* < 0.001). In median, PA occurred 66.5 h after surgery. In follow-up, 31% of all the patients with PA showed development of permanent arrhythmia. The incidence of PA after lower GI surgery is comparatively low. Its occurrence, however, seems to have severe implications since it is significantly associated with higher rates of organ failure and in-house mortality. Also, compared to the general population, the development of permanent arrhythmia is significantly higher in patients who developed new-onset PA.

## Introduction

New-onset postoperative arrhythmias (PAs), such as postoperative atrial fibrillation (POAF), represent common and severe events in the perioperative setting after cardiac surgery. Here, the incidence of POAF varies between 20 and 50%^1,2^. For general and visceral surgery, the incidence and impact of PA has not been well described. The existing literature mostly focusses on surgical procedures of the oesophagus with incidence rates ranging from 9 to 23%^3–7^. While the incidence of PA in patients undergoing esophagectomies might also be explained by the thoracic part of this surgery, systemic factors such as imbalance of electrolytes, activation of sympathetic system and hypoxia followed by vasoconstriction of pulmonary veins have also been depicted as relevant pathophysiological factors for the genesis of PA^8^. Furthermore, intravenous fluid management represents a crucial factor on the development of arrhythmias as prior restricted fluid regimens were significantly associated with a reduction of postoperative complications after elective colorectal resections^9^.

In a recent study of our group we evaluated the impact of PA in patients after upper GI surgery including not only esophagectomies but also gastrectomies and pancreatectomies^10^. The analysis shows increased in-house-mortality associated significantly with PA (among other findings).

Now, in the present retrospective study our aim was to fade out surgical procedures causing mechanical manipulation on pericardium, myocardium or nerve fibres of the thoracic vagus nerve causing a potential bias for an increased occurrence of PA perioperatively. Therefore, our focus here is on patients undergoing surgery of lower GI. For this group of patients previous analysis of the occurrence of PA and its impact are rare: A few studies show incidences ranging from 4.4 to 13.7%^11–13^. To evaluate the impact of PA we also performed long-term follow-ups of patients who underwent lower GI surgery and developed PA regarding possible complications of PA, such as permanent arrhythmias or thromboembolic events.

## Methods

### Patient cohort

Inclusion criteria were patient’s age > 18 years, performed surgery (elective or emergency) of lower GI between 2012 and 2018, and > 18 h monitoring after surgery either at intensive care unit (ICU) or intermediate care unit (IMC). Exclusion criteria were pre-existing arrhythmia and/or implanted pacemaker. Our study was approved by the local ethics committee of the University Medical Centre Goettingen (UMG; study number: 14/2/19). Due to the retrospective character of the study the local ethics committee of the University Medical Centre Goettingen approved the informed consent waiver. For those patients, who were included for follow-up, an informed consent was obtained from all subjects.

Surgical procedures were grouped according to the anatomical localization or type of surgery/indication as following: small bowel resections, ileocecal resections, (extended) right hemicolectomies and resections of the transverse colon, (extended) left hemicolectomies and resections of the sigmoid colon, (sub)total colectomies and proctocolectomies, anterior rectal resections and Hartmann´s procedures, resection rectopexies, low anterior rectal resections, abdominoperineal rectal resections or exenterations, bowel resections after mesenteric ischaemia, multivisceral resections, and cytoreductive surgery (CRS) combined with hyperthermal intraperitoneal chemotherapy (HIPEC).

Among the existing patient data we screened for new-onset PA as well as pre-existing conditions and perioperative complications as multiple cardiovascular risk factors (defined as existence of more than one cardiovascular associated disease, including heart failure, coronary vessel disease, thrombosis, embolism, and prior performed cardiac surgery/intervention), surgical complications (defined as intraoperatively accidentally injured main vessels or organs and/or blood transfusions during surgery), anastomosis and stump insufficiency (diagnosed via rectoscopy or CT scan), wound healing deficit (diagnosed by physician and documented in release papers), Chylus/pancreatic/biliary fistula (diagnosed by physician and documented in release papers), revision surgery, postoperative myocardial infarction, organ failure (diagnosed by physician and documented in release papers), electrolyte disorders (distinct electrolyte shift that required substitution or treatment in general) , postoperative deep vein thrombosis (diagnosed via ultrasound or CT scan), infections (defined as elevated leucocytes or CRP levels with diagnosis of pneumonia, cholangitis, urinary tract infection, or wound infection, during the first 10 days after surgery), sepsis (diagnosed by physician and documented in release papers) and usage of beta blocker in premedication (for blood pressure regulation).

All patients were observed with an ECG monitor device during surgery and after surgery on ICU and IMC. Observation with an ECG monitor device at the wards had to be performed at least for 18 h postoperatively. Monitoring started with induction of anesthesia and stopped with discharge from ICU or intermediate unit. PA was confirmed by ECG and interpreted by experienced intensive care physicians. Atrial fibrillation (AF) was defined following the *ESC Guidelines for the diagnosis and management of atrial fibrillation (2020)* as the minimum duration of an ECG tracing of AF required to establish the diagnosis of clinical AF is at least 30 s. Electrocardiographic characteristics of AF include irregular R-R intervals, absence of distinct repeating P waves and irregular atrial activations. Automatically reports were not used for the study, all arrhythmias were detected by physicians using the criteria of the ESC guidelines. A 12-lead ECG was performed regularly as confirmation if AF was detected on ECG monitor device. Retrospectively we used the documented ICD-10 codes and diagnosis.

Long-term follow-up was obtained from patients who developed PA to screen for permanent arrhythmia, thromboembolic events and overall survival (OS). A standardized questionnaire was used by contacting patients and their general practitioners or cardiologists to ascertain these parameters (Supplementary Fig. 1 shows the translated version of the German questionnaire). Follow-up was performed between January and May 2021.

### Statistical analysis

Comparisons were performed using the Mann–Whitney U test for continuous variables and the chi-square test or Fisher´s exact test for categorical variables.

Multivariable logistic regression models were developed using the 10:1 rule^14^ to confirm the independent associations between postoperative surgical complications and the occurrence of PA, as well as between these postoperative surgical complications and in-house mortality. All selected variables for the regression models were significantly associated with the occurrence of PA (*p* < 0.05) and in-house mortality, respectively. Surgical complications showing significant associations in the uni-variable tests (*p* < 0.05) and with sufficient case numbers (15 for the model of PA and 10 for the model of in-house mortality) were set as independent variables, while the occurrence of PA (or in-house mortality) was set as a dependable variable. Odds ratios (ORs) are presented with 95% confidence intervals. The survival of PA patients who developed a permanent arrhythmia and those who did not was visualized by a Kaplan–Meier plot and compared using the log-rank test.

Statistical analysis was performed using R software (ver. 4.0.2.; R Foundation for Statistical Computing, Vienna, Austria). The global significance level was set to α = 5%.

All methods were performed in accordance with the relevant guidelines and regulations.

Our retrospective analysis follows the Strengthening the Reporting of Observational Studies in Epidemiology (STROBE) reporting guideline for observational studies^15^.

## Results

### Collection of patients

Altogether, n = 1458 patients who underwent surgery of the lower gastrointestinal (GI) tract at the Department of General, Visceral, and Paediatric Surgery of the University Medical Centre Goettingen, Germany between 2012 and 2018 were screened by retrospective chart analysis. Due to pre-existing arrhythmia or a prior implanted pacemaker in the patient history, n = 287 (19.7%) patients were excluded from further analysis in this study. In total, n = 1171 patients were enrolled in our retrospective analysis. All surgical procedures as well as all general demographic and clinical data including pre-existing cardiovascular diseases and consecutive perioperative risk factors, such as hypertension, diabetes, coronary heart disease, heart failure, valvular heart disease, peripheral artery occlusive disease, myocardial infarction, or coagulopathies are listed in Table 1.

**Table 1:** Demographic and clinical data of all enrolled patients.

Parameter	Level	Total	Male	Female
n		1171	612	559
Age	Mean ± sd	64 ± 14	63 ± 13	63 ± 15
	Median (min; max)	65 (18; 96)	64 (18; 96)	65 (18; 95)
Malign diagnosis		703 (60.0%)	381 (62.3%)	322 (57.6%)
Surgery	Surgery for mesenteric ischaemia	26 (2.2%)	16 (2.6%)	10 (1.8%)
	HIPEC in multivisceral resection	18 (1.5%)	6 (1.0%)	12 (2.1%)
	(Extended) right hemicolectomy, transverse colon resection	185 (15.8%)	89 (14.5%)	96 (17.2%)
	(Sub-)total colectomy, proctocolectomy	35 (3.0%)	17 (2.8%)	18 (3.2%)
	Multivisceral resections	92 (7.9%)	34 (5.6%)	58 (10.4%)
	Rectal extirpation/exenteration	91 (7.8%)	57 (9.3%)	34 (6.1%)
	(Extended) left hemicolectomy, sigma resection, anterior rectal resection, Hartmann’s procedure	418 (35.7%)	217 (35.5%)	201 (36.0%)
	Low anterior rectal resection	216 (18.4%)	140 (22.9%)	76 (13.6%)
	Ileocecal resection	65 (5.6%)	32 (5.2%)	33 (5.9%)
	Small bowel resection	8 (0.7%)	3 (0.5%)	5 (0.9%)
	Resection rectopexy	17 (1.5%)	1 (0.2%)	16 (2.9%)
Hypertension		625 (53.4%)	332 (54.2%)	293 (52.4%)
Diabetes mellitus	Type I/II	140 (11.2%)	85 (13.9%)	55 (9.8%)
	Metabolic syndrome	57 (4.7%)	33 (5.4%)	24 (4.3%)
Venous thrombosis (preoperative)		67 (5.7%)	28 (4.6%)	39 (7.0%)
Coronary heart disease		135 (11.5%)	96 (15.7%)	39 (7.0%)
Embolic event		82 (7.0%)	52 (8.5%)	30 (5.4%)
Myocardial infarction (preoperative)		67 (5.7%)	54 (8.8%)	13 (5.4%)
Heart failure		50 (4.3%)	33 (5.4%)	17 (3.0%)
Valvular heart disease		57 (4.9%)	28 (4.6%)	29 (5.2%)
Coagulopathies		19 (1.6%)	5 (0.8%)	14 (2.5%)
Multiple cardiovascular diseases (≥ 2)		357 (30.5%)	214 (35.0%)	143 (25.6%)
Performed cardiac treatment		105 (9.0%)	79 (12.9%)	26 (4.7%)
Premedication	Anticoagulants	299 (25.5%)	184 (30.1%)	115 (20.6%)
	Antiarrhythmics	355 (30.3%)	188 (30.7%)	167 (29.9%)
	Diuretics	316 (27.0%)	165 (27.0%)	151 (27.0%)
	Ras-inhibitors	474 (40.5%)	259 (42.3%)	215 (38.5%)
	Oral antidiabetics	137 (11.7%)	96 (15.7%)	41 (7.3%)

### Incidence of PA

After undergoing surgery of lower GI new-onset PA was seen in n = 56 patients (4.8%), of whom some patients partly developed several types arrhythmia. All detected types of arrhythmia are listed in Table 2.

**Table 2:** Postoperative complications and PA.

Parameter	Level	Total	Male	Female
Surgical complications (intraoperative)		76 (6.5%)	41 (6.7%)	35 (6.3%)
Anastomosis-/stump insufficiency		99 (8.5%)	61 (10.0%)	38 (6.8%)
Wound healing deficit/burst abdomen		260 (22.2%)	142 (23.2%)	118 (21.1%)
Chyle/pancreatic fistula, bile leakage		38 (3.2%)	23 (3.8%)	15 (2.7%)
Myocardial infarction (postoperative)		3 (0.3%)	3 (0.5%)	0 (0.0%)
Revision surgery		180 (15.4%)	94 (15.4%)	86 (15.4%)
Organic failure		70 (6.0%)	39 (6.4%)	31 (5.5%)
Electrolyte disorders		259 (22.1%)	122 (20.0%)	137 (24.5%)
Thrombosis (postoperative)		15 (1.3%)	6 (1.0%)	9 (1.6%)
Infections		156 (13.3%)	95 (15.5%)	61 (10.9%)
Sepsis		66 (5.6%)	34 (5.6%)	32 (5.7%)
Postoperative arrhythmia	Bradycardic atrial fibrillation	3		
	Normofrequent atrial fibrillation	3		
	Tachycardic atrial fibrillation	42		
	Atrial flutter	2		
	Second-degree and third-degree atrioventricular block	3		
	Pulseless electrical activity	2		
	Asystole	5		
	Ventricular fibrillation	1		
	Ventricular tachycardia	1		
	Arrhythmia with cardiopulmonal resuscitation	1		

In our study, the occurrence of PA was very heterogeneous regarding the different types of abdominal surgery.

The highest incidence of PA was seen in patients with bowel resection due to mesenteric ischaemia (7/26; 26.92%), followed by CRS and HIPEC (3/18; 16.67%).

**Figure 1:**
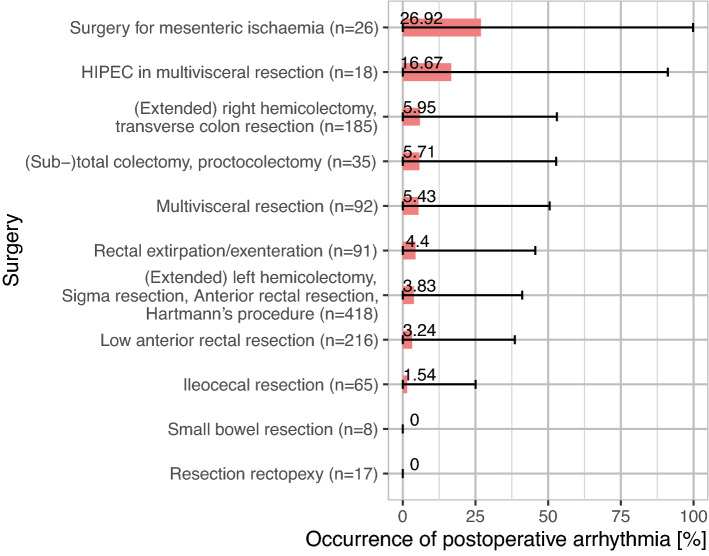
Incidence of postoperative arrhythmia in different types of lower gastrointestinal surgery.

The incidence of PA was distinctively lower in abdominoperineal rectal resections/exenterations (4/91; 4.4%) and in (extended) left hemicolectomies/sigmoid resections/anterior rectal resections and Hartmann´s procedures (16/418; 3.83%). PA was not seen in resections of the small bowel (0%) or resection rectopexies (0%). All surgical procedures and their percentages of arrhythmias are illustrated in Fig. 1.

The median time span between surgery and occurrence of PA was 66.5 h (IQR = 21.0–144.6).

### Risk constellations of developing PA

**Figure 2:**
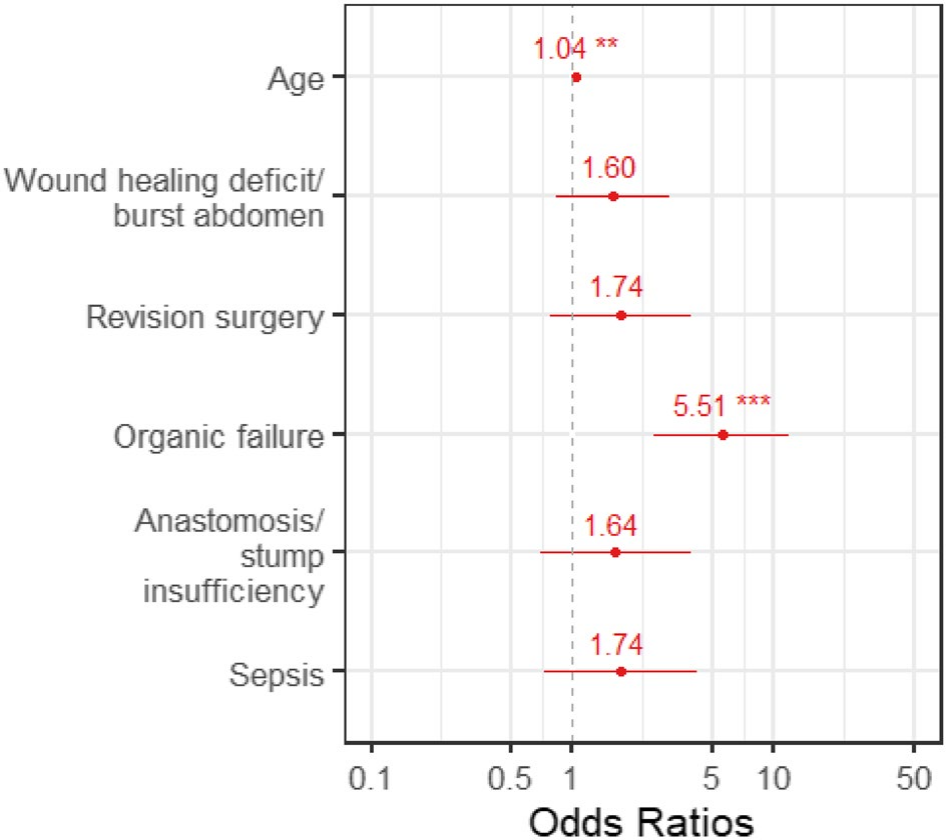
Variables associated with PA as part of the logistic regression model with their odds ratios, *p*-values (* =  < 0.05, ** =  < 0.01, *** =  < 0.001) and 95% confidence interval.

Our study showed a significant association between the occurrence of PA and age of the patients. The median age in the PA group was 72 years (IQR 63–78) versus 64 years (IQR 55–73.5) in the non-PA group (*p* < 0.001). The strongest predictor for the development of PA using multiple testing was organic failure (odds ratio (OR) = 4.62, 95% CI 2.11–10.11, *p* < 0.001), followed by sepsis (OR = 1.88, 95% CI 0.81–4.40, *p* < 0.001), revision surgery (OR = 1.87, 95% CI 0.93–3.74, *p* = 0.08), and multiple pre-existing cardiovascular diseases (OR = 1.62, 95% CI 0.91–2.89, *p* = 0.07; Fig. 2). Table 3 shows all variables that are part of the logistic regression model for the development of postoperative arrhythmias with their odds ratios, *p*-values and 95% confidence interval.

**Table 3:** Presentation of the univariate analyses that are part of the logistic regression model for occurrence of postoperative arrhythmia with *p*-values.

Univariate analysis	*p*-value
Multiple cardiovascular risk factors	0.005**
Surgical complications	0.024*
Anastomosis and stump insufficiency	0.001**
Wound healing deficit	0.0002***
Chylus/pancreatic/biliary fistula	0.42
Revision surgery	< 0.0001***
Postoperative myocardial infarction	0.13
Organ failure	< 0.0001***
Electrolyte disorders	0.002**
Postoperative deep vein thrombosis	0.004**
Infections	0.004**
Sepsis	< 0.0001***
Beta blocker in premedication	0.014*

### PA and its short-term impact

Patients who suffered from PA had a significantly longer stay in the ICU or IMC (median 15 days, IQR 5–25 days versus median 2 days, IQR 1–5 days; *p* < 0.001). Furthermore, the occurrence of PA was significantly associated with increased postoperative in-house mortality as an independent variable in the logistic regression model (16.07% vs. 1.88%; OR: 3.37, 95% CI 1.23–9.28, *p* < 0.001). Other significantly associated variables for in-house-mortality were infections (OR = 3.89, 95% CI 1.60–9.49, *p* < 0.05) and sepsis as the strongest predictor (OR = 27.63, 95% CI 11.90–64.16, *p* < 0.001). Supplementary Table 1 is a presentation of the variables that are part of the logistic regression model for in-house mortality with their odds ratios, *p*-values and 95% confidence interval.

### PA and its long-term impact

For n = 56 patients with newly diagnosed PA follow-up was performed as described above. Follow-up was performed between January and May 2021. Overall median follow-up time was 32, 5 months (IQR = 6.0–69.3). Longest follow-up was 110 months.

**Figure 3:**
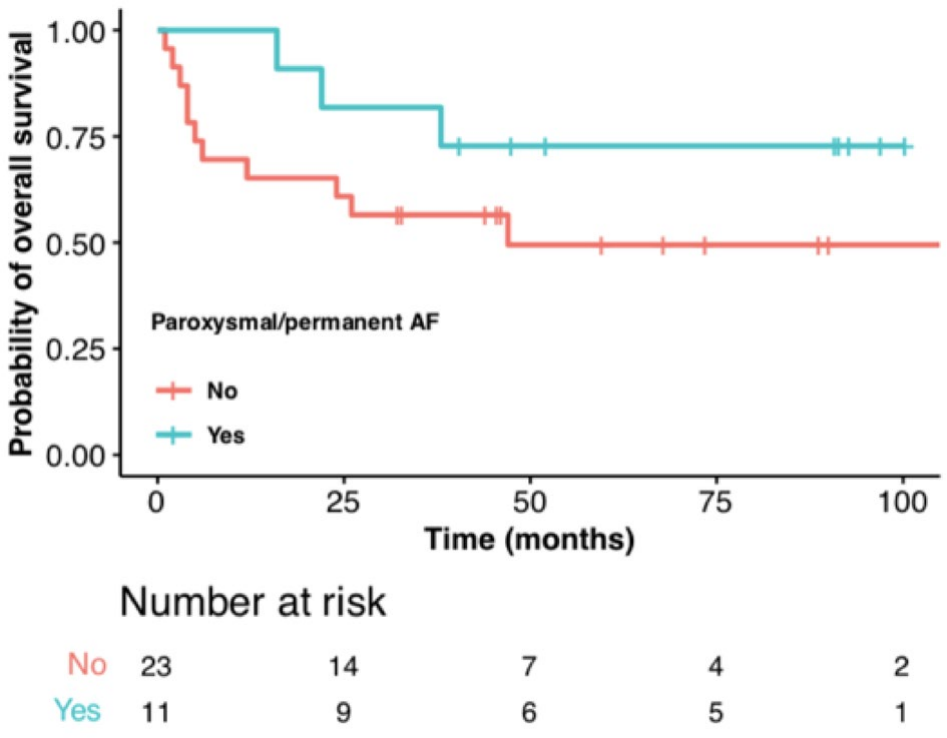
Kaplan–Meier-Curve of patients with PA who developed paroxysmal/permanent arrhythmia (blue) and those who did not develop paroxysmal/permanent PA (red). No significant difference in overall survival was obtained.

In total, 9/56 patients had died during their hospital stay. In n = 11 cases no reliable follow-up data was available (20% loss to follow-up). In n = 36 cases sufficient and reliable follow-up data could be obtained. Among those n = 11 patients (31%) had developed paroxysmal or permanent arrhythmia after discharge. Regarding survival analysis, there was no significant difference in the restricted mean survival time (up to 110 months after surgery) between patients who developed paroxysmal/permanent arrhythmia (86.9 months) and those who did not (61.5 months; log-rank test *p* = 0.2; see Fig. 3).

## Discussion

In the existing literature incidence of new-onset PA after lower GI surgery was reported between 4.4% and 13.7%^11–13^. Kazaure et al.^16^ did a database query of n = 46,716 patients and showed an incidence of atrial fibrillation (AF) of 5.7% after general surgery in patients aged ≥ 55 years. In the present study our result of the analysis of n = 1171 patients showed an incidence of 4.8%, which is in line with the existing data. Although the incidence is comparably low (compared to cardiothoracic surgery or surgery of upper GI), it might have serious consequences: New-onset PA was significantly associated with prolonged stay on ICU and an independent factor significantly associated with increased in-house mortality.

The aetiology of new-onset PA remains unclear and presumably multifactorial. In upper GI surgery, specifically in oesophageal resection with an incidence between 16.5 and 23.1%^4–7^, mechanical manipulation of the myocardium or pericardium is discussed as an important factor. Our previous analysis which investigated the occurrence of PA in patients who underwent upper GI surgery revealed a general incidence of 8.3%^10^. In lower GI surgery different factors are supposed to trigger PA such as an increase in hormonal and sympathetic activity. It results as a stress response due to anaesthesia and surgery^17,18^. Furthermore, surgical trauma and the extent of surgery are suspected to generate postoperative inflammatory processes. For interleukin 6 and 8, that are known to be proinflammatory cytokines, Wu et al. demonstrated an increased expression in peritoneal fluid after colon surgery^19^. In our study, PA was significantly associated with sepsis and infection, which may underline the hypothesis of an infection associated cause of PA. Both factors, PA and sepsis, were associated with increased in-house-mortality. However, according to our multivariable regression model the association of both factors with in-house-mortality was independent from each other. If the inflammatory process or other postoperative complications are the cause of PA cannot be addressed in this retrospective study.

Another potential important factor for the occurrence of PA might be the perioperative intravenous fluid management, which was previously described as one relevant part being associated with an increase of weight and cardiopulmonary complications^9^. Intravenous fluid volumes also affected secretion levels of N-terminal-pro-brain natriuretic peptide (NT-pro-BNP) in patients who underwent elective colorectal surgery being predictive regarding cardiopulmonary complications^20^. Due to the retrospective character of the present study, we do not have valid data of the amount of perioperative intravenous fluid regimens to address this possible confounder. As a general rule, we tend to be restrictive with intravenous fluid substitution after colorectal surgery in our clinic.

Concerning the time point of the development of PA in our study, the median occurrence of PA was 66.5 h (IQR = 21.0–144.6) after surgery, which is in concordance with a previous analysis claiming a peak of arrhythmias within the first four days postoperatively^13,21^. The wide range of occurrence may further strengthen the inflammatory association rather than the fact of a strong correlation to surgery as inflammatory processes may also started later during the hospital stay. Furthermore, this time frame could lead to the assumption that potential arrhythmias of patients being discharged from ICU or IMC treatment in the early postoperative phase (postoperative days 1 (POD 1) and 2 (POD2)) might remain undetected at a normal ward where there is no continuous monitoring. The vast majority of our patients who underwent lower GI surgery were transferred to a normal ward earlier than 66.5 h after surgery. Therefore, we assume that the number of unreported events of PA in the perioperative setting is considerably higher.

Regarding the distribution of arrhythmias in our study, the highest incidence was found in mesenteric ischaemia (26.92%). As mesenteric ischaemia mostly occurs due to other pre-existing diseases, particularly because of diverse arteriosclerotic risk factors, many of those patients are expected to suffer from severe heart and vessel diseases^22,23^. Furthermore, electrolyte imbalances, increased lactate and an increased inflow of inflammatory cytokines occur due to manipulation during surgery. These factors severely impact the occurrence of PA. Interestingly, CRS combined with simultaneous application of HIPEC (16.67%) showed a high percentage of PA. In general, CRS and simultaneous HIPEC include surgery that takes several hours followed by 90 min of hyperthermic (42 °C/107.6 °F) chemotherapy diluted in sodium chloride 0.9%. The duration of this surgery as well as the fact that a volume of up to 5 to 7 L of diluted chemotherapy passes both the abdominal cave and the oesophageal hiatus entering the mediastinum might have an effect on the development of PA. This might be associated with the analysis of Brandstrup et al. showing that the amount of i.v.-fluid can possibly cause cardiac complications^20^.

In our analysis, when comparing the incidence of PA after right-side hemicolectomy to abdominoperineal rectal resections/exenterations, with the latter causing presumably a significantly larger surgical trauma, the incidence of PA after right-side hemicolectomy was slightly higher (5.95% vs. 4.4%). Therefore, other factors than the pure surgical trauma have to be taken into account. Most likely age and co-morbidities may play a pivotal role explaining the difference. Additionally, the individual risk of each patient respecting their medical histories is another predisposition for the risk of PA^24–26^.

In this study, the occurrence of PA was associated with a higher rate of development of permanent/paroxysmal arrhythmia: Overall 31% of all patients who developed PA and had sufficient follow-up data showed paroxysmal or permanent arrhythmia after discharge, whereas the incidence of AF in a study with an age-matched population is 0.8%^27^.

However, overall survival did not significantly differ between those patients who developed paroxysmal or permanent arrhythmia after PA and those who did not. We believe that this might be blurred by the relevance of other factors such as oncological prognosis. Surprisingly, those patients who developed a permanent arrhythmia after PA even seem to have a better long-term outcome (although the difference is not significant). One explanation could be that since no standard cardiological surveillance was performed after the occurrence of PA, patients received adequate treatment when PA was detected and patients where PA was not detected did not receive adequate treatment. However, it is a small set of patients in the follow-up group and the difference between the two groups (patients with detected paroxysmal/permanent arrhythmia versus patients without diagnosis of paroxysmal or permanent arrhythmia) remains statistically insignificant.

The presented study has certain limitations: The retrospective origin of the analysis does not allow any conclusion about the causalities. Also, the sequence of PA and the appearance of surgical and non-surgical complications have not been documented. Plus, the real incidence of PA after lower GI surgery might be higher because patients generally leave ICU or IMC treatment on the first days after surgery. Hence, a prospective study with close monitoring of patients during the first week after surgery would be of great importance. Additionally, cardiological follow-up after discharge of patients with PA would be desirable. Nevertheless, to the best of our knowledge, this study includes the largest cohort to date detecting the occurrence of PA in patients who underwent lower GI surgery and clearly demonstrated PA as an important risk factor for prolonged ICU stay, increased in-house mortality after surgery and higher risk for development of paroxysmal/permanent arrhythmia after discharge.

## Supplementary Information

Supplementary Table 1.

Supplementary Figure 1.

## Data Availability

Drs. Rühlmann and Azizian had full access to all of the data in the study and take responsibility for the integrity of the data and the accuracy of the data analysis. The datasets used and/or analysed during the current study are available from the corresponding author on reasonable request.
