# Supplementary material for: Incidence and impact of new-onset postoperative arrhythmia after surgery of the lower gastrointestinal tract
Source: Sci Rep. 2023 Jan 23;13:1284. doi: 10.1038/s41598-023-27508-4 (PMC9870894; doi:10.1038/s41598-023-27508-4)
Supplement: Supplementary file 1 — Supplementary Table 1. [file 41598_2023_27508_MOESM1_ESM.docx]

| **Univariate analysis** |  | **p-value** |
| --- | --- | --- |
| Multiple cardiovascular risk factors |  | 0.010* |
| Surgical complications |  | 0.010* |
| Anastomosis and stump insufficiency |  | 0.102 |
| Wound healing deficit |  | 0.207 |
| Chylus / pancreatic /biliary fistula |  | 0.070 |
| Revision surgery |  | <0.0001*** |
| Postoperative myocardial infarction |  | 1 |
| Organ failure |  | <0.0001*** |
| Electrolyte disorders |  | 0.002** |
| Postoperative deep vein thrombosis |  | 1 |
| Infections |  | 0.0009*** |
| Sepsis |  | <0.0001*** |
| Medication of antiarrhythmics on Intensive Care Unit |  | <0.0001*** |
| Age |  | 0.064 |
| Postoperative arrhythmia |  | <0.0001*** |
| Supplementary Table 1: Presentation of the univariate analyses that are part of the logistic regression model for mortality with p-values | | |
