# Supplementary material for: Incidence and impact of new-onset postoperative arrhythmia after surgery of the lower gastrointestinal tract
Source: Sci Rep. 2023 Jan 23;13:1284. doi: 10.1038/s41598-023-27508-4 (PMC9870894; doi:10.1038/s41598-023-27508-4)
Supplement: Supplementary file 2 — Supplementary Figure 1. [file 41598_2023_27508_MOESM2_ESM.doc]

**English version oft the patients‘ survey used**

| Universitätsmedizin Göttingen |  | Klinik für Allgemein-, Viszeral- und Kinderchirurgie  Direktor: Univ.-Prof. Dr. med. Michael Ghadimi |
| --- | --- | --- |
| Allgemein-, Viszeral- und Kinderchirurgie, Univ.-Prof. Dr. med. Michael Ghadimi  Robert-Koch-Str. 40, 37075 Göttingen |  |  |
| **Survey**  Please mark applicable answer! |  |  |
|  |  | 37099 Göttingen **Briefpost**  Robert-Koch-Straße 40, 37075 Göttingen **Adresse**  0551 39-66104 **Telefon**  0551 39-66106 **Fax**  info@chirurgie-goettingen.de **E-Mail** |

Göttingen, January 2021

1) Which year the surgery at UMG was performed?

| 2012 | 2013 | 2014 | 2015 | 2016 | 2017 | 2018 |
| --- | --- | --- | --- | --- | --- | --- |

2) Which organ was the surgery performed on?

| Esophagus | Gaster | Colon | Liver | Pancreas | Adrenal gland | other |
| --- | --- | --- | --- | --- | --- | --- |

3) Were any arrhythmia known before surgery?

| Yes (type?) |  | No |
| --- | --- | --- |

4) Was any arrhythmia diagnosed after discharge from hospital (UMG)?

| Yes (type?) |  | No |
| --- | --- | --- |

If yes, after what time period did the arrhythmia occur?

| Still in hospital | During first 4 weeks after discharge | During first 3 months after discharge | During first 6 months after discharge | During first year after discharge | After fist year after discharge |
| --- | --- | --- | --- | --- | --- |

5) Did you developed a stroke after discharge?

| Ja |  | Nein |
| --- | --- | --- |

If yes, after what time period did the stroke occur?

| Still in hospital | During first 4 weeks after discharge | During first 3 months after discharge | During first 6 months after discharge | During first year after discharge | After fist year after discharge |
| --- | --- | --- | --- | --- | --- |

6) Do you take any blood-thinning medication?

| Yes (what sort of medication?) |  | no |
| --- | --- | --- |
